# GCN2 is required to maintain core body temperature in mice during acute cold

**DOI:** 10.1152/ajpendo.00181.2023

**Published:** 2023-10-04

**Authors:** Jordan L. Levy, Emily T. Mirek, Esther M. Rodriguez, Brian Zalma, Jeffrey Burns, William O. Jonsson, Harini Sampath, Kirk A. Staschke, Ronald C. Wek, Tracy G. Anthony

**Affiliations:** ^1^Department of Nutritional Sciences, New Jersey Institute for Food, Nutrition and Health, Rutgers University, New Brunswick, New Jersey, United States; ^2^Department of Biochemistry and Molecular Biology, Indiana University School of Medicine, Indianapolis, Indiana, United States; ^3^Indiana University Melvin and Bren Simon Comprehensive Cancer Center, Indianapolis, Indiana, United States

**Keywords:** activating transcription factor 4 (ATF4), energy expenditure, eukaryotic initiation factor 2 (eIF2), hypothermia, mechanistic target of rapamycin complex 1 (mTORC1)

## Abstract

Nonshivering thermogenesis in rodents requires macronutrients to fuel the generation of heat during hypothermic conditions. In this study, we examined the role of the nutrient sensing kinase, general control nonderepressible 2 (GCN2) in directing adaptive thermogenesis during acute cold exposure in mice. We hypothesized that GCN2 is required for adaptation to acute cold stress via activation of the integrated stress response (ISR) resulting in liver production of FGF21 and increased amino acid transport to support nonshivering thermogenesis. In alignment with our hypothesis, female and male mice lacking GCN2 failed to adequately increase energy expenditure and veered into torpor. Mice administered a small molecule inhibitor of GCN2 were also profoundly intolerant to acute cold stress. *Gcn2* deletion also impeded liver-derived FGF21 but in males only. Within the brown adipose tissue (BAT), acute cold exposure increased ISR activation and its transcriptional execution in males and females. RNA sequencing in BAT identified transcripts that encode actomyosin mechanics and transmembrane transport as requiring GCN2 during cold exposure. These transcripts included class II myosin heavy chain and amino acid transporters, critical for maximal thermogenesis during cold stress. Importantly, *Gcn2* deletion corresponded with higher circulating amino acids and lower intracellular amino acids in the BAT during cold stress. In conclusion, we identify a sex-independent role for GCN2 activation to support adaptive thermogenesis via uptake of amino acids into brown adipose.

**NEW & NOTEWORTHY** This paper details the discovery that GCN2 activation is required in both male and female mice to maintain core body temperature during acute cold exposure. The results point to a novel role for GCN2 in supporting adaptive thermogenesis via amino acid transport and actomyosin mechanics in brown adipose tissue.

## Graphical abstract

**Figure:**
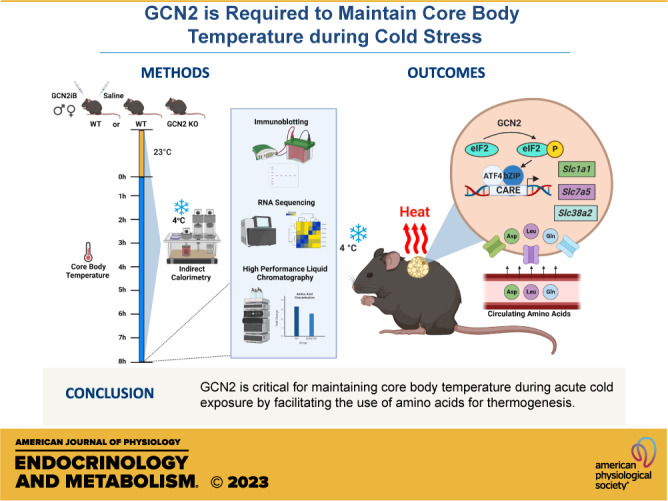

## INTRODUCTION

On acute cold exposure, thermal balance is maintained by activating behavioral and physiological responses. These adaptive responses serve to decrease heat loss and increase metabolic heat production to protect against a potentially dangerous drop in core body temperature known as hypothermia (1, 2). Mammals can increase heat generation by the mechanical contraction of skeletal muscle and through nonshivering thermogenic processes, which uncouple mitochondrial cellular respiration from ATP production (3).

The mechanisms that support nonshivering thermogenesis are not fully understood. On cold stimulation, sympathetic release of norepinephrine enhances the levels and activity of uncoupling protein 1 (UCP1) protein in brown adipocytes, resulting in the release of protons across the inner mitochondrial membrane to generate heat (4, 5). Free fatty acids are recognized as primary drivers of proton conductance through UCP1 in brown adipose; however, other mechanisms in brown adipose exist such as altered redox metabolism, futile consumption of ATP, and UCP1-independent proton leak (6). In rodents, UCP1-mediated nonshivering thermogenesis is activated by feeding low protein or sulfur amino acid restricted diets, resulting in leanness (7, 8). These reports have attracted scientific and public attention for their clinical potential as a modifier of body weight in addition to their application for therapeutic hypothermia and defense against secondary hypothermia (9, 10). The fundamental processes underlying adaptive thermogenesis is thus an area of study with practical application to many pathophysiological conditions.

The integrated stress response (ISR) is a cellular adaptation and survival mechanism activated by various environmental stressors. On the sensing of a particular stress condition, one or more ISR protein kinases phosphorylate the alpha subunit of eukaryotic initiation factor 2 (eIF2). This phosphorylation event lowers global protein synthesis while concomitantly favoring the synthesis of proteins, which function to promote adaptive gene expression (11). The ISR is implicated as a regulator of nonshivering thermogenesis in BAT (12, 13) because ISR gene targets such as fibroblast growth factor 21 (FGF21) and solute carrier (SLC) proteins are essential for maintaining core body temperature during acute cold exposure (14, 15). Under various models of amino acid insufficiency, the activation of the eIF2 kinase, general control nonderepressible 2 (GCN2) is required for the early hepatic production of FGF21 (7, 16–20). In addition, loss of GCN2 activity impedes SLC-mediated inward transport of amino acids during nutrient stress (21). Whether or not GCN2 is activated by cold stress in mammals and to what extent GCN2 influences FGF21 and SLC responses during cold remain unknown.

The objective of this study was to determine the role of GCN2 in the maintenance of core body temperature during cold exposure in mice. We hypothesized that GCN2 is required for adaptation to acute cold stress via activation of the ISR, resulting in liver production of FGF21 and increased amino acid transport to support nonshivering thermogenesis. We found that loss of GCN2 activity both genetically and pharmacologically rendered male and female mice severely cold intolerant. Contrary to our hypothesis, the production of FGF21 in liver or brown adipose did not correlate with maximal induction of adaptive thermogenesis. UCP1 and other key genes involved in adaptive thermogenesis in brown adipose were also not differentially responsive by genotype or sex. Instead, actomyosin-mediated tension and SLC-mediated transmembrane transport were the top biological processes negatively impacted by loss of GCN2. Loss of GCN2 impeded cold-induced transcriptional increases in amino acid transporters in BAT which corresponded with higher circulating amino acid concentrations and lower intracellular amino acid concentrations in BAT. These findings suggest a novel role for GCN2 in guiding the use of amino acids for energy and mediating actomyosin mechanics to support thermogenic capacity of brown adipose during cold stress.

## MATERIALS AND METHODS

### Animal Model

The animal procedures and experimental protocols were approved by the Institutional Animal Care and Use Committees at Rutgers, The State University of New Jersey. Adult (12–23 wk old) male and female wildtype (WT) and whole body *Gcn2*^−/−^ mice (GCN2 KO) on the C57Bl/6J genetic background for 10 generations were used. All mice were bred at the Bartlett animal facility on the Rutgers University Cook campus. Before the studies, all mice had free access to food (5001 laboratory rodent diet; LabDiet) and purified water and were maintained on a 12-h light-dark cycle at 23°C with same-sex littermates until experimental group assignment, wherein mice were housed in individual plastic cages with soft bedding. Before cold exposure, animals were individually housed and kept at room temperature (RT; 23°C) for a 5-day acclimation period and subsequently exposed to 4°C (Cold). For drug administrations, GCN2iB (30 mg/kg), or vehicle (05% methylcellulose and 5% DMSO in H_2_O) was administered 1 h before cold exposure (21).

### Group Assignment and Blinding

WT and GCN2 KO animals were randomly assigned to experimental treatment groups. Following group assignments, animals were deidentified using alphanumerical coding.

### Cold Experiments

Following the 5-day single housing acclimation period at room temperature (23°C), WT male and female mice were transferred to individual plastic cages with no bedding located in a refrigerated room held at 4°C starting at Zeitgeiber Time (ZT) 1. The mice were held at 4°C for 8 h and killed at ZT9. The tissues collected at ZT9 were used in all serum and tissue analyses reported throughout the manuscript. One experiment was conducted in male mice only that started at ZT13 and ended at ZT21. Because these male mice were killed at a different time of day, their tissues were not used in subsequent analyses. Animals were provided with free access to food and water over the full duration of the study period.

### Sample Collection and Storage

Male and female mice were killed by decapitation without the use of anesthesia. Unless otherwise specified, all tissue and serum samples were harvested at the end of the cold exposure period. Following dissection, tissue samples were immediately frozen in liquid nitrogen and stored at −80°C. All subsequent biochemical analyses were performed using samples collected during the light cycle (Zeitgeiber Time 09).

### Comprehensive Laboratory Animal Monitoring System

Mice were placed within the Columbus Instruments Comprehensive Lab Animal Monitoring System (CLAMS, Columbus Instruments, Columbus, OH). The system features 12 cages placed within an enclosed environment-controlled cabinet. Each cage provides real-time monitoring of horizontal and vertical activity, wheel activity, feeding and drinking, oxygen consumption, and CO_2_ production every 13 min. These measurements were recorded over a 3-day period. In the first 48 h, the animals were kept at room temperature, followed by a period in which the temperature of the environment-controlled cabinet descended to 4°C (∼2 h) and then held at 4°C for the remaining duration of the study. CLAMS data were placed into the CalR application (22) where the data were compiled into hourly measurements for further analyses.

### Core Body Temperature

Core body temperatures were assessed using a handheld probe (Kent Scientific) to record rectal temperatures over time.

### Tissue Glycogen

To measure glycogen levels, deionized water was added to 10–15 mg of frozen, powdered gastrocnemius and plantaris or liver tissue at a 1:20 ratio and quickly homogenized on ice using plastic pestles in standard 1.5-mL microcentrifuge tubes. Samples were then clarified by centrifugation at 18,000 *g* for 10 min at 4°C. The supernatant was collected, and glycogen levels were measured by a Glycogen Assay Kit (Abcam, ab65620) according to the manufacturer’s instructions.

### Serum Nonesterified Fatty Acids

Serum free fatty acid levels were measured using a colorimetric kit (Abcam, ab65341) according to the manufacturer’s instructions.

### Serum Triglycerides

Serum triglyceride content was measured immediately following decapitation with a handheld meter and test strips (Polymer Technology Systems; CardioChek).

### Real-Time RT-qPCR

Total RNA was isolated using the Direct-zol RNA MiniPrep Plus Kit (Zymo Research) following instructions provided by the manufacturer. The quality of the isolated RNA was measured by determining the A260/280 and A260/230 ratios using a Nanodrop fluorospectrometer (Thermo Fisher Scientific). Integrity of the isolated RNA was further assessed by visualization of the rRNA on agarose gels. Reverse transcription was performed with 1 μg of each RNA sample using the High-Capacity cDNA Reverse Transcription Kit (Thermo Fisher, Cat. No. 4368814) according to the manufacturer’s instructions. The resulting cDNA was used for quantitative analysis of gene expression with a SYBR green system using a StepOne real-time PCR system (Applied Biosystems). Primer sequences are listed in Supplemental Table S1 and each were previously reported or generated using the NCBI Primer-Blast tool. All primers were verified by testing primer efficiency and specificity using the standard curve method and melt curve analysis, respectively. Levels of gene transcripts in samples were measured in technical duplicates or triplicates within 96-well plates and normalized to β-actin. Comparisons were made using the 2^−ΔΔCt^ method and then expressed as fold-change relative to the WT mice at room temperature.

### RNA Sequencing

Isolated RNA was quantified using a Qubit 2.0 Fluorometer (ThermoFisher Scientific), and RNA integrity was verified using the TapeStation System (Agilent Technologies). RNA sequencing libraries were prepared using the NEBNext Ultra II RNA Library Prep Kit for Illumina (New England Biolabs) following the manufacturer’s instructions. Briefly, mRNAs were initially enriched with Oligo d(T) beads. Enriched mRNAs were fragmented for 15 min at 94°C. First-strand and second-strand cDNAs were subsequently synthesized. cDNA fragments were end-repaired and adenylated at 3' ends and universal adapters were ligated to cDNA fragments, followed by index addition and library enrichment by PCR with limited cycles. The sequencing libraries were validated with the TapeStation System (Agilent Technologies) and quantified by using Qubit 2.0 Fluorometer (ThermoFisher Scientific). Further validation of the libraries was carried out by quantitative PCR (KAPA Biosystems) of selected genes.

The sequencing libraries were multiplexed and clustered onto a flowcell. After clustering, the flowcell was loaded onto the Illumina HiSeq instrument according to manufacturer’s instructions. The samples were sequenced using a 2 × 150 base pair Paired-End configuration. Image analysis and base calling were conducted by the HiSeq Control Software. Raw sequence data (.bcl files) generated from Illumina HiSeq were converted into fastq files and demultiplexed using Illumina bcl2fastq 2.20 software. One mismatch was allowed for index sequence identification. After investigating the quality of the raw data, sequence reads were trimmed to remove possible adapter sequences and nucleotides with poor quality using Trimmomatic v.0.36. The trimmed reads were mapped to the *Mus musculus* reference genome available on ENSEMBL using the STAR aligner v.2.5.2b. BAM files were generated by this step. Unique gene hit counts were calculated by using feature Counts from the Subread package v.1.5.2. Only unique reads that fell within exon regions were counted. After extraction of gene hit counts, the gene hit counts table was used for downstream differential expression analysis. Using DESeq2 (23), a comparison of gene expression between the groups of samples was performed. The Wald test (24) was used to generate *P* values and Log2 fold-changes. Genes with adjusted *P* values < 0.05 and absolute (log2 fold-changes) >1 were deemed as differentially expressed between treatment groups. Gene ontology analyses were performed on the differentially expressed set of genes using The Reactome Knowledgebase (25), Gene Ontology (GO): Biological Process tool (26) and Categorizer (27).

### SDS-PAGE and Immunoblotting

Protein lysates were prepared from frozen tissue pulverized using a mortar and pestle under liquid nitrogen. Approximately 20 mg of pulverized frozen tissue was suspending in ice cold RIPA lysis solution consisting of 25 mM HEPES, 2 mM EDTA, 10 mM DTT, 50 mM sodium fluoride, 50 mM β-glycerophosphate pentahydrate, 3 mM benzamidine, 1 mM sodium orthovanadate, 0.5% (wt/vol) sodium DOC, 1% (wt/vol) SDS, 1× protease inhibitor cocktail (P8340, Millipore-Sigma), and 5 nM microcystin (33893, Millipore-Sigma). The homogenized lysates were subjected to centrifugation for clarification at 10,000 *g* for 10 min at 4°C. Equal amounts of total protein were mixed 1:1 (vol:vol) with a 2 × sample buffer solution [20% (vol/vol) glycerol, 60 mM Tris (pH 6.8), 2% (wt/vol) SDS, 0.01% (wt/vol) bromophenol blue, and 5% (vol/vol) β-mercaptoethanol], after which samples were heated at 95°C for 4 min and then stored at −80°C until further use. Gel electrophoresis was performed by separating equal amounts of protein (as determined using Pierce BCA Protein Assay, 23227, Thermo Fisher Scientific) by electrophoresis in SDS-polyacrylamide gels and separated proteins were then electrotransferred onto PVDF membranes. Protein-bound membranes were blocked for 1 h at room temperature before incubation with primary antibodies overnight. Immunoreactive bands were visualized by first incubating the membranes for 1 h at room temperature with appropriate secondary antibodies (Supplemental Table S2), followed by briefly incubating the membranes with enhanced chemiluminescence (ECL) solution (RPN2235, Cytvia Amersham ECL Select Western Blotting Detection Agent, Cytvia, Marlborough, MA) to image the targeted proteins (FluorChem M, ProteinSimple, San Jose, CA). All antibodies used were available commercially and validated by the respective manufacturer. Densitometry was performed using ImageJ (Fiji, v. 1.0).34. Values were normalized to either total protein using Ponceau staining or to the total of the respective proteins phosphorylated and unphosphorylated species and expressed as fold-change compared with the WT animals kept at 23°C for each respective sex.

### Serum FGF21

Serum concentrations of FGF21 were measured using a commercially available sandwich enzyme-linked immunosorbent assay (ELISA; RD291108200R; BioVendor LLC). The ELISA assay was performed according to the manufacturer’s instructions as previously reported (18).

### Serum and Intracellular Amino Acid Concentrations

Serum and intracellular tissue amino acid concentrations were analyzed using high performance liquid chromatography (HPLC), as previously described (28). Serum and intracellular proteins were precipitated and removed by adding serum or tissue to 0.1% formic acid in methanol at a 1:3 ratio and vortexed. Tissue samples were centrifuged at 10,000 *g* for 10 min at 4°C, and the supernatant was collected. Serum and tissue samples were filtered using Captiva nondrip lipid filtration tubes (Agilent Cat. No. A5400635). The recovered eluate in the amount of 20 μL was spiked with 5 μL of internal standard (equimolar mix of sarcosine and norvaline, 0.1 mM each) and analyzed using reversed-phase HPLC (Agilent 1200 HPLC instrument) according to the manufacturer’s protocol (Agilent Publication; Cat. No. 5990-4547EN). Briefly, the separation of each amino acid was carried out using a gradient mix of mobile phase A (10 mM Na_2_HPO_4_, 10 mM Na_2_B_4_O_7_, 5 mM NaN_3_, pH 8.2) and mobile phase B (acetonitrile/methanol/water = 45/45/10) on ZORBAX Eclipse Plus C18 column at the speed of 0.42 mL/min. To allow for amino acid detection by fluorescence, derivatization was done automatically using an autosampler according to the manufacturer’s protocol, which consisted of two consequent steps: *1*) conjugation to o-phthalaldehyde and *2*) conjugation to fluorenylmethyloxycarbonyl chloride. Standard curves for each amino acid were retrieved from analyzing a set of equimolar standard mixtures. A total of 10 standard mixtures were used ranging from 2.25 to 900 μM of each amino acid. Analysis of chromatograms was performed using Agilent OpenLAB software. Tissue and serum amino acid concentrations are listed in Supplemental Tables S3–S6.

### Statistical Analysis

Analyses and visualization were performed using R version 4.2.2 (29) and RStudio. The following packages were used: tidyverse, ggpubr, DescTools. A Wilkes Shapiro test was used to check data for normality. Data that did not follow a normal distribution were normalized using a log10 transformation. Data were analyzed using either a GLM, Student’s *t* test or a two-way ANOVA followed by a post hoc analysis using a Tukey’s HSD correction for multiple comparisons. Comparisons among groups with different sample sizes were conducted using the Kruskal–Wallis nonparametric test. Power analysis was conducted to inform on effect size using the G*Power 3.1.9.7 application (30).

## RESULTS

### Loss of GCN2 Impedes the Thermogenic Response to Acute Cold Exposure

**Figure 1.**
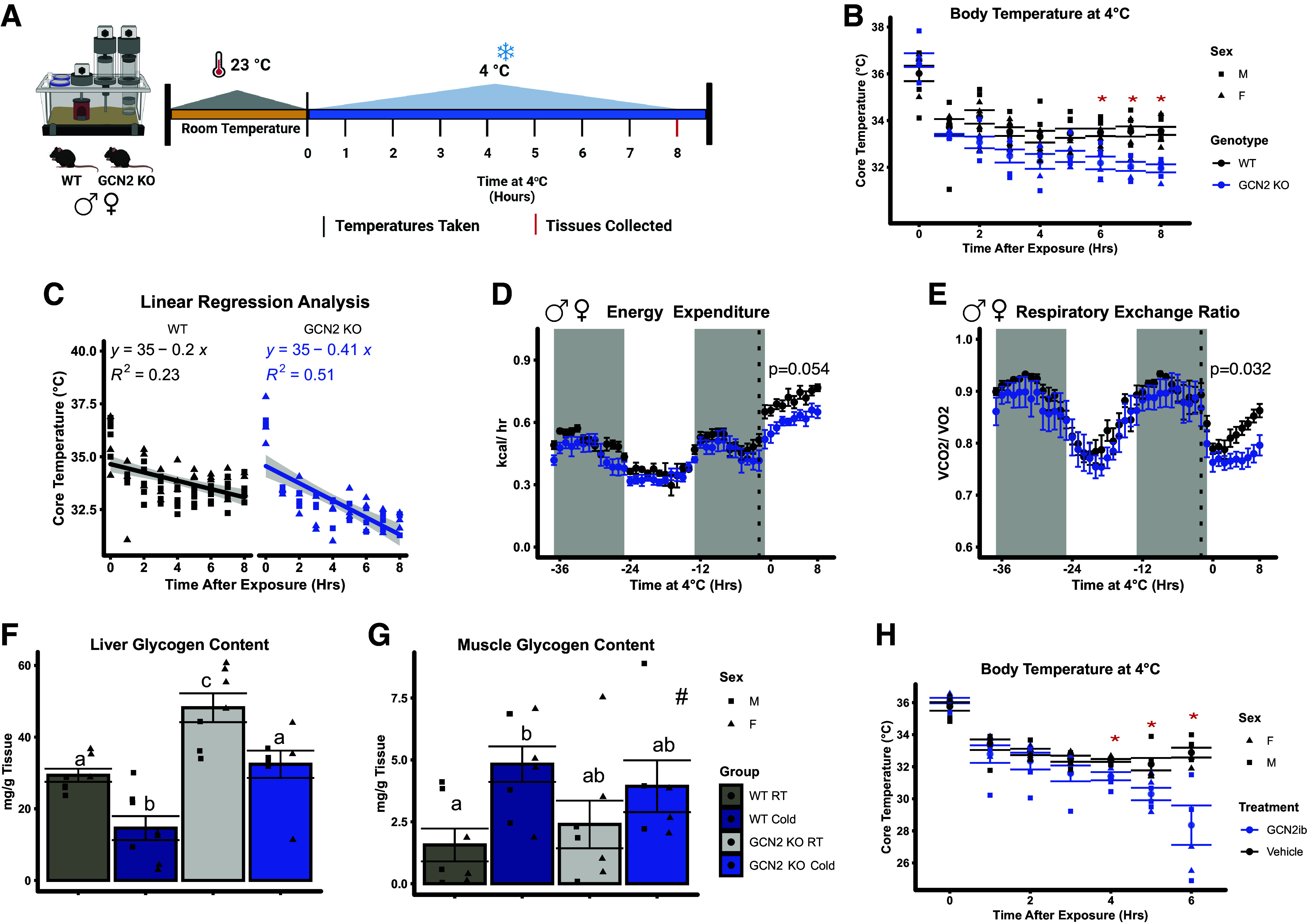
Loss of GCN2 impedes the thermogenic response to acute cold exposure. *A:* visual representation of the experimental design. *B:* hourly rectal temperatures of male and female wild type (WT) and *Gcn2* knockout (GCN2 KO) mice housed at 4°C for 8 h during the light cycle. *C*: linear regression analysis of the core body temperature of WT and GCN2 KO mice over the 8 h cold exposure period. Whole body energy expenditure (*D*) and respiratory exchange ratio (*E*) of male and female WT and GCN2 KO mice. The dotted vertical line represents the time at which the temperature in the CLAMS began to descend to 4°C. The gray backdrop represents the time at which the lights were off in the CLAMS. *P* values represent the results of our statistical analyses performed during the acute exposure period. Liver (*F*) and skeletal muscle (*G*) glycogen content of mice held at 23°C room temperature (RT) or following 8 h exposure to 4°C (Cold) during the light cycle. *H*: hourly rectal temperatures of GCN2iB (30 mg/kg) or vehicle treated male and female WT mice housed at 4°C for 6 h during the light cycle. Rectal temperature measurements are shown as individual data points ± SE for each respective group and timepoint. CLAMS data are expressed as means ± SE. Bar chart values are presented as means ± SE with individual data points overlaid. The shape of the individual datapoints in *B*, *C*, and *F*–*H* denotes the sex of the mice. CLAMS data in *D* were analyzed using a General Linear Model in CalR. All other data were analyzed using a two-factor ANOVA followed by a Tukey’s post hoc test. #Main effect of temperature, *P* < 0.05. *Main effect of genotype, *P* < 0.05. Groups not sharing a common letter are different and indicate a statistical interaction. *n* = 6–8 male and female mice per group. Panel *A* was created with BioRender.

To assess the role of GCN2 in maintaining core body temperature during acute cold exposure, we placed freely fed room temperature (RT)-reared male and female WT and GCN2 KO mice at 4°C (Cold) beginning at the start of their light cycle and recorded rectal temperatures hourly for 8 h (Fig. 1*A*). No differences in initial body weight or final tissue weights according to genotype were noted at the end of the study (Supplemental Fig. S1, *A*–*D*). Following 6 h of cold exposure, all GCN2 KO mice displayed lower core body temperatures as compared with WT mice (Fig. 1*B*, Supplemental Fig. S2, *A* and *B*). A linear regression analysis revealed that, based on the slope of the line of best fit, the core body temperature of GCN2 KO mice decreased at approximately twice the rate of WT mice (Fig. 1*C*). In conjunction with these findings, a rise in hourly energy expenditure (EE; Fig. 1*D*), regardless of biological sex (Supplemental Fig. S2, *C* and *D*), and a rise in the respiratory exchange ratio (RER; Fig. 1*E*), also independent of biological sex (Supplemental Fig. S2, *E* and *F*), were both stalled in GCN2 KO as compared with WT mice during cold exposure. Physical activity and food intake were similar between the genotypes in both male and female mice (Supplemental Fig. S2, *G*–*J*).

To determine if the lower RER in GCN2 KO mice during cold was due to differences in glucose utilization, we measured glycogen content in liver and skeletal muscle. Both genotypes showed significant reductions in liver glycogen following cold exposure, although the liver glycogen content of GCN2 KO mice was significantly higher than WT mice in both RT and cold conditions (Fig. 1*F*). In contrast to the liver, skeletal muscle glycogen content was increased as compared with RT independent of genotype (Fig. 1*G*). Literature reports postulate that higher muscle glycogen content following cold stress is due to increased glucose uptake into skeletal muscle independent of insulin action (31, 32). To examine if the lower RER in GCN2 KO during cold was due to altered lipid utilization, we measured triglycerides and nonesterified fatty acids in serum. GCN2 deletion did not impact serum triglyceride concentrations (Supplemental Fig. S2*K*) or serum nonesterified fatty acid levels (Supplemental Fig. S2*L*) in cold-stressed mice. We interpret these measurements to suggest that altered utilization of glycogen may contribute to but do not fully explain the stalled RER in cold-stressed GCN2 KO mice.

To address the contribution of feeding status to the observed cold intolerance in GCN2 KO mice, we then exposed male WT and GCN2 KO mice to 4°C beginning at the start of their dark cycle. During cold exposure, both genotypes reduced their overall food intake (Supplemental Fig. S2*M*) and ambulatory activity (Supplemental Fig. S2*N*). Similar to the previous cold stress experiments, GCN2 KO mice displayed significantly lower energy expenditure (Supplemental Fig. S2*O*) and slightly reduced RER (Supplemental Fig. S2*P*) as compared with WT mice. Notably, GCN2 KO mice entered a torpor-like state at 6 h, causing the experiment to end early.

To further confirm whether GCN2 kinase activity is required for adaptive thermogenesis, WT mice were treated with GCN2iB, a specific ATP competitive inhibitor of GCN2 kinase activity (33), or vehicle at the start of the light cycle and then subjected to cold (4°C) challenge. Similar to GCN2 KO, pharmacological inhibition of GCN2 rendered both female and male mice cold intolerant, with core body temperatures significantly declining by 4 h and becoming severely hypothermic after 6 h, requiring early termination of the experiment (Fig. 1*H*). Immunoblot analysis showed that GCN2iB inhibited GCN2 phosphorylation in liver (Supplemental Fig. S3*A*) and BAT (Supplemental Fig. S3*B*). These experiments confirm that GCN2 activity is required for acute cold tolerance.

### GCN2 Is Required for Cold-Induced Hepatic FGF21 Secretion in Male Mice Only

**Figure 2.**
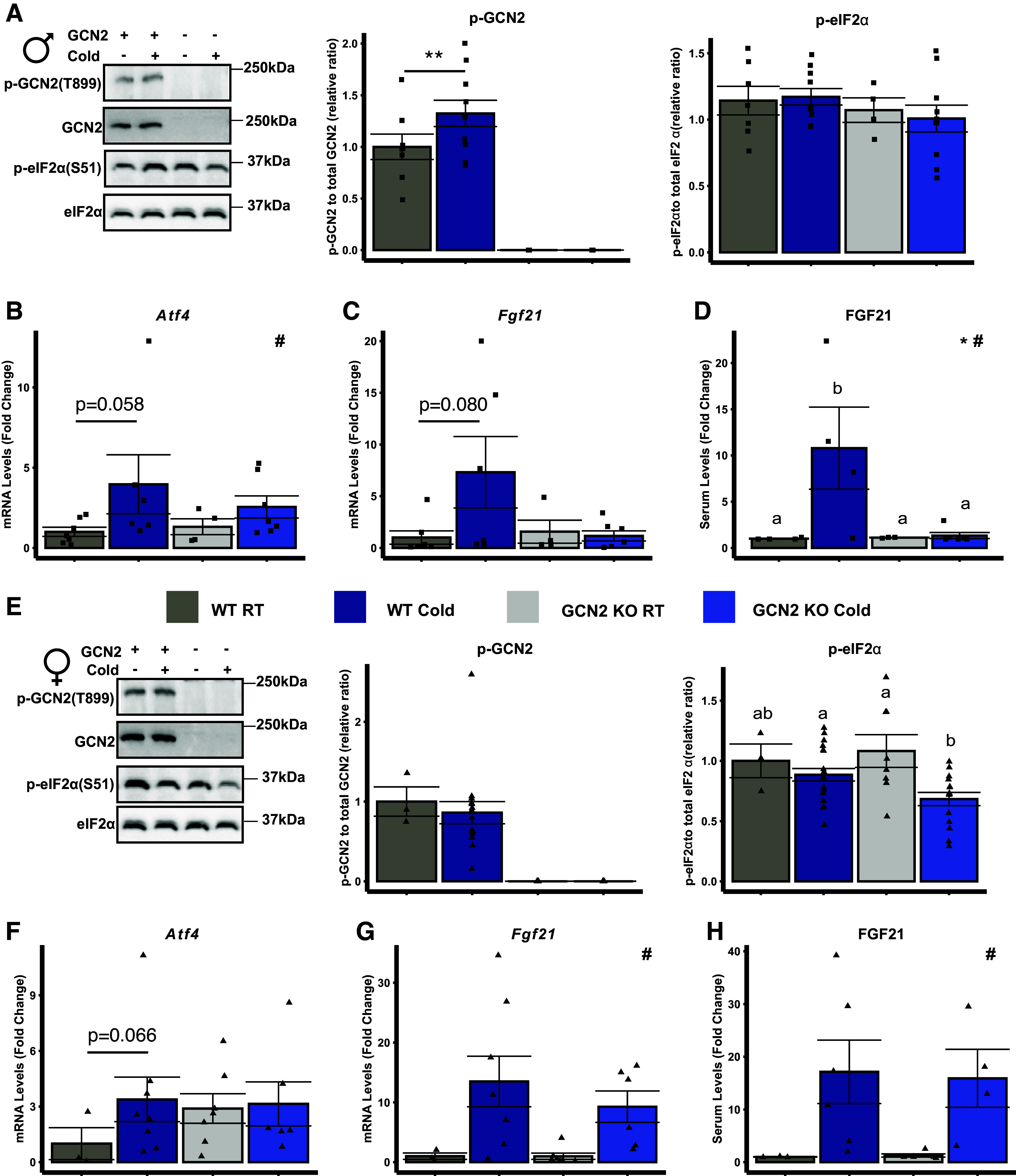
GCN2 is required for cold-induced hepatic FGF21 secretion in male mice only. *A:* representative immunoblots and ratios of hepatic phosphorylated GCN2 and eIF2α relative to their respective total protein in male wild type (WT) and *Gcn2* knockout (GCN2 KO) mice held at 23°C room temperature (RT) or following 8 h exposure to 4°C (Cold) during the light cycle. + and – signify the presence or absence of GCN2 or Cold. Hepatic mRNA concentrations of *Atf4* (*B*) and *Fgf21* (*C*) in male mice at the end of the experiment. *D*: normalized serum FGF21 levels in male mice at the end of the experiment. *E*: representative immunoblots and ratios of hepatic phosphorylated GCN2 and eIF2α to their respective total protein in female WT and GCN2 KO mice held at RT or following 8 h of Cold during the light cycle. + and – signify the presence of absence of GCN2 or Cold. Hepatic mRNA concentrations of *Atf4* (*F*) and *Fgf21* (*G*) in female mice at the end of the experiment. *H*: normalized serum FGF21 levels in female mice at the end of the experiment. All values are expressed relative to the WT RT group. Serum FGF21 values ranged from 18 to 1,500 pg/mL. GCN2 phosphorylation was analyzed by Student’s *t* test, **P < 0.05. Data were analyzed by two-factor ANOVA or Kruskal–Wallis test followed by a Tukey’s post hoc test. *Main effect of genotype, *P* < 0.05; #main effect of temperature, *P* < 0.05. Groups not sharing a common letter indicate a statistically significant interaction, *P* < 0.05. Bar chart values are presented as means ± SE with individual data points overlaid. *n* = 3–8 mice per group.

GCN2 is necessary for hepatic FGF21 secretion during acute dietary forms of amino acid insufficiency, and liver-derived FGF21 is required for thermogenesis in mice subjected to acute cold stress (14, 16, 34). Based on these literature reports, we hypothesized that GCN2 is necessary for activation of the ISR and FGF21 production during acute cold exposure. In WT male mice, phosphorylation of GCN2 but not eIF2α increased in liver following 8 h of cold (Fig. 2*A*). GCN2 was required for increased mRNA levels of the ISR core effector, activating transcription factor 4 (*Atf4*; Fig. 2*B*) and the ATF4 target gene, *Atf5* (Supplemental Fig. S3*C*), during cold exposure whereas other ISR target genes, *Gadd34* and *Ddit3*, were unchanged (Supplemental Fig. S3, *D* and *E*). In addition, GCN2 was required for cold-induced increases in hepatic *Fgf21* mRNA levels (Fig. 2*C*) and serum FGF21 concentrations (Fig. 2*D*) in males. In comparison, WT females following 8 h of cold exposure displayed no change in the phosphorylation of hepatic GCN2 or eIF2α but mice lacking *Gcn2* showed loss of eIF2α phosphorylation with cold (Fig. 2*E*). GCN2-dependent changes in hepatic *Atf4* (Fig. 2*F*) and *Atf5* (Supplemental Fig. S3*C*) but not *Gadd34* and *Ddit3* were noted in females during cold (Supplemental Fig. S3, *D* and *E*). Cold-stimulated hepatic *Fgf21* mRNA levels (Fig. 2*G*) and serum FGF21 concentrations (Fig. 2*H*) were GCN2 independent in females. Neither males nor females showed PERK activation in liver during cold stress (Supplementary Fig. S3*F*). These data demonstrate a sex-specific role for GCN2 in mediating hepatic FGF21 production under hypothermic conditions.

### ISR Activation Does Not Directly Regulate Uncoupling-Mediated Thermogenesis in Brown Adipose Tissue during Acute Cold Exposure

**Figure 3.**
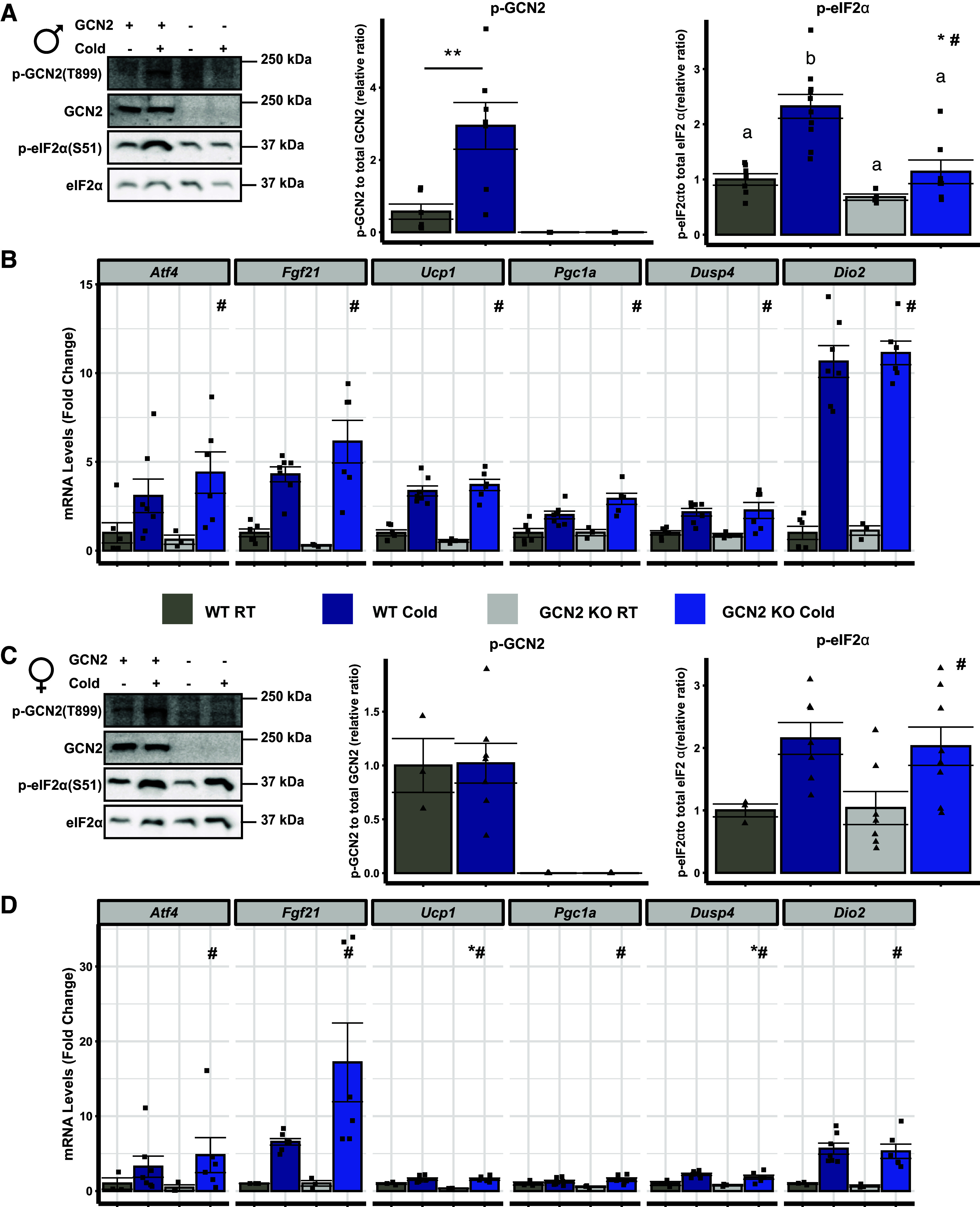
ISR activation does not directly regulate nonshivering thermogenesis in brown adipose tissue during acute cold exposure. *A:* representative immunoblots and relative ratios of phosphorylated GCN2 and eIF2α to their respective total protein in brown adipose tissue (BAT) from male wild type (WT) and *Gcn2* knockout (GCN2 KO) mice held at 23°C room temperature (RT) or following 8 h exposure to 4°C (Cold) during their light cycle. + and – signify the presence of absence of GCN2 or Cold. *B*: relative mRNA levels of *Atf4*, *Fgf21*, *Ucp1*, *Ppargc1a*, *Dusp4*, and *Dio2* in the BAT of male mice. *C*: representative immunoblots and ratios of phosphorylated GCN2 and eIF2α relative to their respective total protein in BAT samples of female WT and GCN2 KO mice held at RT or following Cold during their light cycle. + and – signify the presence of absence of GCN2 or Cold. *D*: BAT mRNA concentrations of *Atf4*, *Fgf21*, *Ucp1*, *Ppargc1a*, *Dusp4*, and *Dio2* in female mice. All values are expressed relative to WT RT. GCN2 phosphorylation was analyzed by Student’s *t* test, **P < 0.05. All other data were analyzed by two-factor ANOVA or Kruskal–Wallis test followed by a Tukey’s post hoc test. *Main effect of genotype, *P* < 0.05; #main effect of temperature, *P* < 0.05. Groups not sharing a common letter indicate a statistically significant interaction, *P* < 0.05. Bar chart values are presented as means ± SE with individual data points overlaid. *n* = 3–8 mice per group.

A few reports suggest that cold exposure may execute an ISR transcriptional program in BAT (12, 36). To build on this, we examined ISR activation in BAT following cold stress. We observed that in both females and males, PERK phosphorylation did not change according to temperature or genotype (Supplementary Fig. S3*G*). In male mice, cold exposure increased GCN2 and eIF2α phosphorylation (Fig. 3*A*). Interestingly, GCN2-dependent ISR activation in the BAT of cold-stressed males coexisted with GCN2-independent increases in *Atf4* and *Fgf21* (Fig. 3*B*). Furthermore, thermogenic genes associated with FGF21 including *Ucp1*, *Pgc1a*, *Dusp4*, and *Dio2* (Fig. 3*B*) as well as hormone sensitive lipase phosphorylation and UCP1 protein levels (Supplemental Fig. S3*H*) were increased in BAT by cold regardless of GCN2 status. In the BAT of cold-stressed female mice, eIF2α phosphorylation increased in both WT and GCN2 KO mice (Fig. 3*C*). Increased mRNA levels of *Atf4* and *Fgf21* were also noted in the BAT of WT and GCN2 KO female mice exposed to cold (Fig. 3*D*). Similar to males, acute cold exposure increased mRNA abundance of thermogenic genes (Fig. 3*D*) and hormone sensitive lipase activation and UCP1 protein levels (Supplemental Fig. S3*H*) in BAT of females regardless of GCN2 status. These data lead us to conclude that ISR activation does not directly regulate nonshivering thermogenesis in BAT during acute cold exposure.

### GCN2 Is Required for Upregulation of SLC Transporters and Myosin Heavy Chain Genes in BAT during Acute Cold Exposure

**Figure 4.**
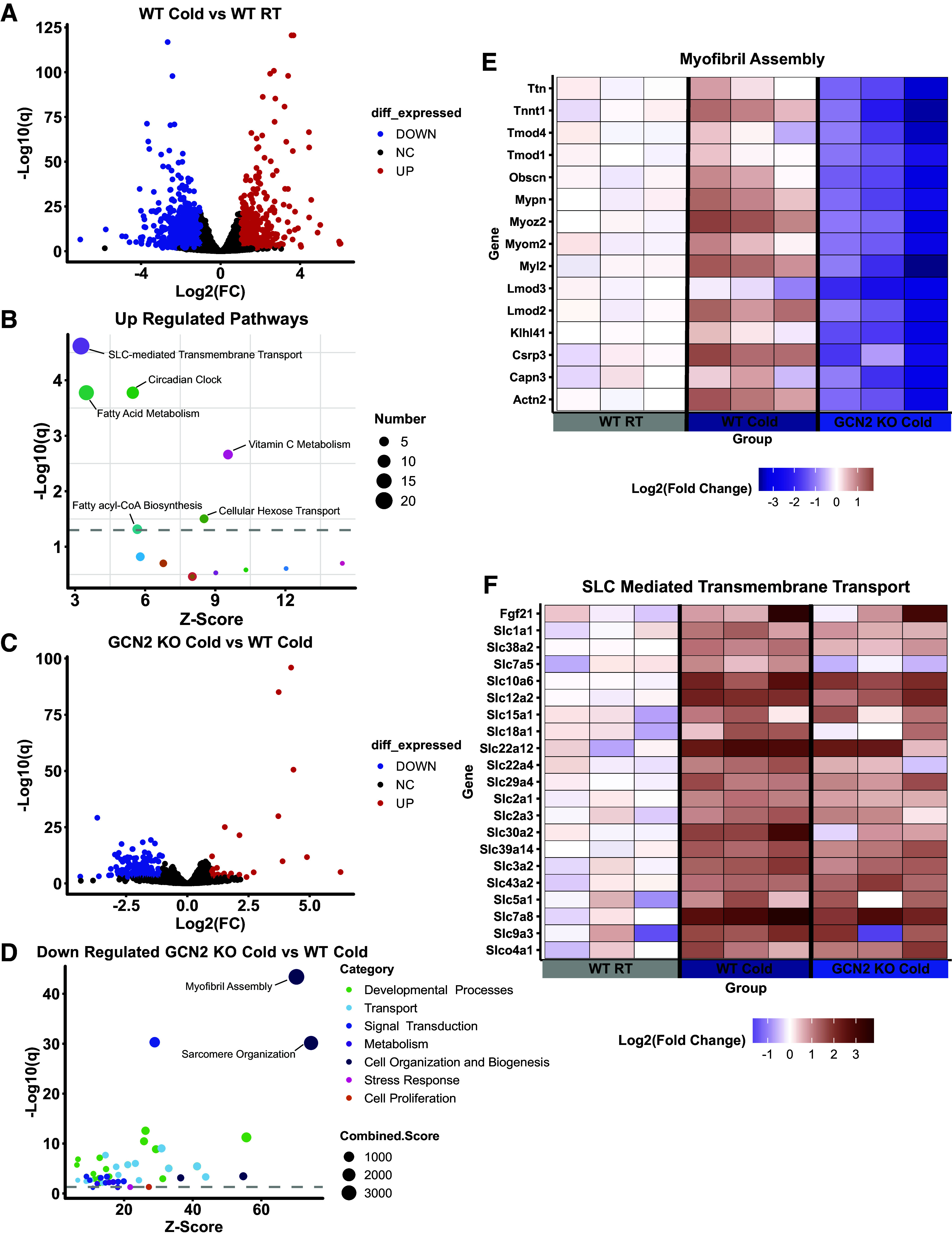
GCN2 is required for upregulation of SLC transporters and myosin heavy chain genes in bat during acute cold exposure. *A:* volcano plot illustrating differentially expressed transcripts from the brown adipose tissue of wild type (WT) mice following 8 h exposure to 4**°**C (Cold) as compared with mice held at 23**°**C room temperature (RT). The scatterplot represents statistical significance (−log10(*q*)] vs. magnitude of change [log2(FC) on *x*-axis]. The dots represent individual genes. Black dots represent unchanged (NC) genes. Blue dots represent genes that were significantly decreased (DOWN). Red dots represent genes that were significantly increased (UP). The false discovery rate cutoff was *q* < 0.05 and absolute (log2FC)>1. *B*: scatter plot representing significantly upregulated biological pathways in WT Cold mice compared with RT using the Reactome Pathways Database. The size of each circle represents the number of genes within the pathway. The dotted line represents the false discovery rate cutoff, with circles above having a *q* value of <0.05. *C*: volcano plot illustrating differentially expressed transcripts when comparing *Gcn2* knockout (GCN2 KO) Cold mice relative to WT Cold mice. *D*: scatter plot representing significantly downregulated pathways in GCN2 KO Cold mice compared with WT Cold using the Gene Ontology Database. The size of each circle represents the combined score {[−log10(q)] *(z-score)}. The dotted line represents the false discovery rate, with circles above having a *P*-adjusted value (*q*) < 0.05. *E*: heat map illustrating the pathway term Myofibril Assembly in individual samples. For each gene, the log2(fold**-**change) is expressed relative to the average expression of the WT RT group. *F*: heat map illustrating the pathway term SLC**-**Mediated Transmembrane Transport in individual samples. For each gene, the log2(fold**-**change) is expressed relative to the average expression of the WT RT group. *n* = 3 mice per group, groups contain both male and female mice. SLC, solute carrier.

To more fully explore the BAT transcriptome during acute cold stress and determine which biological pathways were altered, we conducted RNA sequencing and then compared transcript abundance in WT male and female mice following 8 h of cold exposure to their RT counterparts (23) (Fig. 4*A*). Differentially expressed upregulated genes were analyzed using The Reactome Knowledgebase (25). Although many of the output terms were involved in lipid metabolism, the upregulated pathway with the lowest *P*-adjusted value (*q*) and the highest number of genes was “SLC-Mediated Transmembrane Transport” (Fig. 4*B*). Many of the genes in this category were ATF4 target genes that function in transmembrane amino acid transport, including *Slc1a1*, *Slc38a2*, and *Slc7a5*.

To determine the impact of GCN2 status and function on cold-induced changes within the BAT transcriptome, we then compared our cold-exposed GCN2 KO mice to our cold-exposed WT mice using the same threshold criteria (Fig. 4*C*). The transcripts found to be significantly downregulated in GCN2 KO were then analyzed using The Reactome Knowledgebase and Gene Ontology (GO): Biological Process tool (26) yielding similar results. The top 125 downregulated GO terms were further classified using the Categorizer tool (27). The four categories with the largest proportion of GO terms associated with GCN2 status were as follows: Developmental processes, Transport, Signal transduction, and Metabolism (Supplemental Fig. S4*A*). We noted that many of the genes found under the Developmental processes category are involved in actin and myosin organization (Fig. 4, *D* and *E*) and include transcripts encoding class II myosin heavy chain proteins, which facilitate tensional responses in cells during cold stress. BAT from cold-exposed GCN2 KO mice displayed significantly lower transcript abundance of *Myh1*, *Myh2*, *Myh4*, *Myh7*, *Myh9*, and *Myh10* (Supplemental Fig. S4*B*), which is interesting because actomyosin-mediated biomechanical signaling is critical for achieving maximal thermogenic capacity in adipocytes (37).

GCN2 is known to be important for the upregulation of SLC transcripts involved in the transport of amino acids and other metabolites. To investigate the impact of GCN2 on the upregulation of cold-induced SLC transcripts in BAT, we compared our GCN2 KO cold-exposed mice to WT mice held at room temperature. Genetic loss of GCN2 impeded the cold-induced upregulation of SLC transcripts including genes encoding the amino acid transporters *Slc38a2*, *Slc7a5*, and *Slc1a1* (Fig. 4*F*). Taken together, our data suggested that GCN2 is required for cold-induced transcriptional upregulation of SLC transcripts encoding genes involved in amino acid uptake within BAT independent of sex.

### GCN2 Is Required to Maintain Intracellular Amino Acid Levels in BAT during Acute Cold Exposure

**Figure 5.**
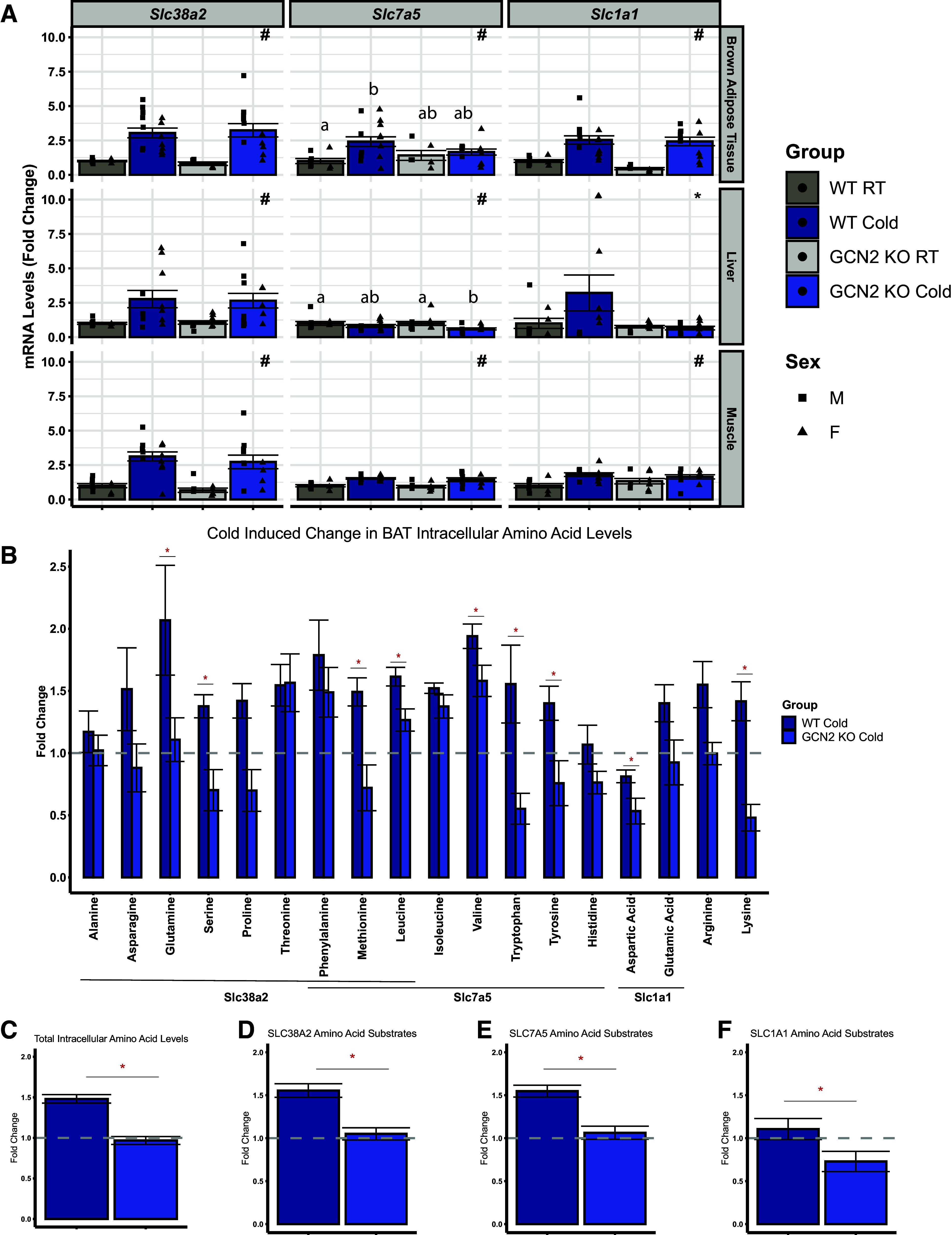
GCN2 is required to maintain intracellular amino acid levels in BAT during acute cold exposure. *A:* relative *Slc38a2*, *Slc7a5*, and *Slc1a1* mRNA levels in the brown adipose tissue (BAT), liver, and muscle (gastrocnemius and plantaris) of male and female wild type (WT) and *Gcn2* knockout (GCN2 KO) mice held at room temperature (RT, 23°C) or following 8 h exposure to 4°C (Cold) during their light cycle. *B*: fold-change in intracellular amino acid levels within the BAT of cold-treated WT and GCN2 KO mice. The lines underneath the *x*-axis span the amino acid substrates for the transporters Slc38a2, Slc7a5, and Slc1a1. *C*: fold-change in total intracellular BAT amino acid levels following 8 h of Cold in WT and GCN2 KO mice. Fold-change of SLC38A2 (*D*), SLC7A5 (*E*), and SLC1A1 (*F*) amino acid substrates following the 8 h of Cold in WT and GCN2 KO Mice. All values are expressed relative to WT RT for each respective amino acid and gene. In *B–F*, the dashed line represents the amino acid levels in WT mice held at RT. Bar chart values are presented as means ± SE with individual datapoints overlayed. mRNA expression data were analyzed by two-factor ANOVA or Kruskal–Wallis test followed by a Tukey’s post hoc test to determine main (genotype, temperature) and interaction effects. *Main effect of genotype, *P* < 0.05; #main effect of temperature, *P* < 0.05. Groups not sharing a common letter indicate a statistically significant interaction, *P* < 0.05. *n* = 7–15 mice per group for qPCR experiments. Intracellular amino acid levels were compared using a one-tailed Student’s *t* test. *n* = 4 mice per group for HPLC analysis. Groups include both male and female mice.

To determine if an impeded upregulation of amino acid transporters in the BAT of GCN2 KO mice during acute cold exposure could influence amino acid uptake, we first measured cold-induced changes in serum amino acid concentrations. In line with previously published work, WT mice showed an overall increase in serum amino acids following 8 h of cold exposure (Supplemental Fig. S5*A* and Supplemental Table S3) (38, 39). However, GCN2 KO mice displayed a higher cold-induced rise in total serum amino acid concentrations as compared with WT mice (Supplemental Fig. S5*B*). We then further categorized our serum amino acids based on the SLC amino acid transporters previously identified in our BAT RNA-seq data set and found that GCN2 KO mice displayed significantly higher concentrations of SLC38A2 and SLC7A5 amino acid substrates in the serum (Supplemental Fig. S5, *C* and *D*) whereas SLC1A1 amino acid substrates did not change (Supplemental Fig. S5*E*). To confirm our findings in our RNA-seq analysis as well as observe if other amino acid utilizing tissues may be affecting these elevated serum amino acid levels, we measured transcript abundance of *Slc38a2*, *Slc7a5*, and *Slc1a1* by RTqPCR in the BAT, liver, and skeletal muscle. Similar to our RNA-seq analysis, *Slc38a2*, *Slc7a5*, and *Slc1a1* mRNA abundances were significantly increased in BAT on cold exposure and the increase in *Slc7a5* required GCN2 (Fig. 5*A*). In contrast, GCN2 was not required for increased *Slc38a2*, *Slc7a5*, and *Slc1a1* transcript abundances in skeletal muscle or liver during cold (Fig. 5*A*). We then measured intracellular amino acid concentrations in BAT (Fig. 5*B* and Supplemental Table S4) and skeletal muscle (Supplemental Fig. S5*F* and Supplemental Table S5). GCN2 KO mice displayed no change in intracellular amino acid concentrations in skeletal muscle but showed significantly lower total intracellular amino acids in BAT (Fig. 5*C*), including substrates for SLC7A5 (Fig. 5*D*), SLC38A1 (Fig. 5*E*), and SLC1A1 (Fig. 5*F*). Finally, to assess whether intracellular reduction in amino acids in BAT altered mechanistic target of rapamycin complex 1 (mTORC1) signaling, we measured changes in the phosphorylation of mTOR, S6K1, and RPS6. Consistent with the published literature, mTORC1 signaling was maintained during acute cold exposure in BAT (40) (Supplemental Fig. S6, *A*–*D*). No significant effects of temperature, genotype, or biological sex were observed. Overall, our data suggest a tissue-specific role of GCN2 in mediating cold-induced uptake of amino acids to support thermogenesis in BAT.

## DISCUSSION

This work shows that GCN2 is necessary to support adaptive thermogenesis during acute cold exposure in mice by increasing amino acid transport into brown adipose. The physiological requirement for GCN2 to maintain core body temperature during acute cold stress is intact in both biological sexes. These data are supported by a number of observations across model systems and experimental designs. In plant seedlings, GCN2 is activated by and promotes adaptation to cold stress (41). In male mice, several ISR target genes including *Atf4*, *Fgf21*, and the SLC class of transporters are linked to the thermogenic response to acute cold exposure (10). Dietary models that induce adaptive thermogenesis, most notably amino acid insufficiency by diet or drug, also report that GCN2 is required for the early and acute hepatic production of FGF21 and for amino acid homeostasis via SLC-mediated transport (12, 16, 21). Taken together in conjunction with our current results, we conclude that GCN2-directed ISR-driven increases in amino acid transport into BAT contributes to the maintenance of core body temperature during cold stress. This work carries broad implications for understanding the regulation of energy expenditure and body temperature under conditions of environmental stress. Considering the emergence of a cadre of GCN2 activators and inhibitors on the market, this work also holds practical implications in the use of these chemical agents for the treatment of chronic diseases such as cancer and in the prevention of secondary hypothermia in patients with compromised thermotolerance such as trauma and surgical patients.

The two primary mechanisms, which defend core body temperature during hypothermic conditions, are thermogenesis (i.e., heat production) and insulation (i.e., heat retention). Impedances in either of these physiological processes under hypothermic conditions lead to a reduction in core body temperature and risk of hypothermia (42). Numerous studies demonstrate the physiological role of endogenous FGF21 on acute cold exposure and its metabolic effects on thermoregulation and metabolic homeostasis (43–46). Our data support a unique role for GCN2 in regulating cold-induced hepatic FGF21 production in male mice independent of eIF2 phosphorylation. This role is also seen during other forms of stress, such as dietary sulfur amino acid insufficiency (18, 19). In addition, similar to previous experimental models, loss of GCN2 activity impedes SLC-mediated inward transport of amino acids (21, 47). It is likely that an impedance in amino acid transport prevents the use of amino acids for oxidation, which is recognized as playing a critical role in the maintenance of thermoregulation in BAT (15).

Following our RNA sequencing analysis within the BAT, the GO category that was found to be the most severely impacted on GCN2 deletion involved both actin and myosin organization and contraction. BAT possesses muscle-like characteristics due to originating from precursor cells that express myogenic factor 5 (48). In addition to expressing muscle-specific genes, the mitochondrial proteome and cytoskeletal structure of brown adipocytes are more reminiscent of myocytes than adipocytes (49–51). Previous work shows that thermogenic adipocytes including brown adipocytes contain an extensive cytoskeletal network that supports thermogenesis by aiding in the shuttling of proteins and nutrients to fuel cellular respiration. Muscle-specific type II myosin heavy chains are a critical component of this cytoskeletal network by facilitating cell tensional responses in cells during cold stress (37). Cold-treated GCN2 KO mice displayed significantly lower transcript abundance of *Myh1*, *Myh2*, *Myh4*, *Myh7*, *Myh9*, and *Myh10*. Previous work shows that GCN2 also engages in important cross talk between cytoskeletal organization and protein synthesis, indirectly sensing changes in F-actin filament organization (52). Future studies examining how GCN2 activity affects actomyosin mechanics are warranted.

Aside from the role of GCN2 in increasing thermogenesis, a secondary role in heat retention during acute cold exposure is worth considering. Loss of skin barrier function is reported following genetic deletion of the ISR gene target stearoyl-CoA desaturase-1 (*Scd1*) either globally or in skin only, rendering both mutants extremely cold intolerant (53, 54). SCD1 functions in the production of monounsaturated fatty acids, which are major components of tissue lipids. Activation of the ISR by amino acid insufficiency or high-fat diet alters *Scd1* mRNA expression; this effect is lost in mice lacking GCN2 (55–57). Although unstudied, the lipid composition of skin may differ in mice lacking GCN2, resulting in reduced heat retention during cold stress. However, the extreme cold sensitivity of WT mice treated with GCN2iB argues against this as a primary contributor. Furthermore, SCD1 deletion significantly increases energy expenditure under cold stress to compensate for the heat loss due to impaired barrier function (53). Another concept worth consideration is compromised skin function, as observed during wound healing in keratinocytes lacking GCN2 (58). In this work, genetic deletion or pharmacological inhibition of GCN2 in keratinocytes delayed collective cell migration and wound closure, with the major effect involving a failure to maintain intracellular free cysteine and a loss of reactive oxygen species coordination at the wound site. Interestingly, this loss corresponded with disruptions in cytoskeletal arrangements and focal adhesion dynamics that were ATF4 independent. Although our energy expenditure data argue that a loss of GCN2 activity impedes thermogenesis, a role for GCN2 activity in skin barrier function is worth exploring in future studies.

Taken together, we conclude that GCN2 function is critical for maintaining core body temperature during acute cold exposure by facilitating the use of amino acids for thermogenesis and supporting cytoskeletal mechanics to achieve maximal thermogenic capacity.

## DATA AVAILABILITY

RNA sequencing data are deposited in GEO, Accession No. GSE234972.

## SUPPLEMENTAL DATA

10.7282/00000350 Supplemental Figs. S1–S6 and Supplemental Tables S1–S6: https://doi.org/10.7282/00000350.

## GRANTS

This work was supported by grants from the National Institutes of Health DK109714 (to T.G.A. and R.C.W.), National Institutes of Health DK126963 (H.S.), R35GM136331 (to R.C.W.), and the Melvin and Bren Simon Comprehensive Cancer Center P30CA082709 (to K.A.S.).

## DISCLOSURES

R.C.W. is a member of the advisory board of HiberCell, Inc. K.A.S. consults for HiberCell, Inc. and Aclaris Therapeutics and receives research support from HiberCell, Inc. T.G.A. consults for HiberCell, Inc. None of the other authors has any conflicts of interest, financial or otherwise, to disclose.

## AUTHOR CONTRIBUTIONS

T.G.A. conceived and designed research; J.L.L., E.T.M., E.M.R., B.Z., J.B., and W.O.J. performed experiments; J.L.L. and H.S. analyzed data; J.L.L., K.A.S., R.C.W., and T.G.A. interpreted results of experiments; J.L.L. prepared figures; J.L.L. and T.G.A. drafted manuscript; J.L.L., E.T.M., B.Z., W.O.J., H.S., K.A.S., R.C.W., and T.G.A. edited and revised manuscript; J.L.L., E.T.M., E.M.R., J.B., W.O.J., H.S., K.A.S., R.C.W., and T.G.A. approved final version of manuscript.
